# Nurses' Actions of Maintaining Patient Dignity: An Integrative Review

**DOI:** 10.1111/scs.70305

**Published:** 2026-08-17

**Authors:** Elina Paala, Sanna Koskinen, Minna Stolt

**Affiliations:** ^1^ Department of Nursing Science University of Turku Turku Finland

**Keywords:** dignity, integrative review, nurses' actions

## Abstract

**Background:**

Dignity is both an objective, intrinsic right and a subjective experience that forms through interaction with other people. Humane care is a precondition for nurses' ethics practice and maintaining patients' dignity. During care, patients' dignity is maintained through nurses' actions. However, acute care settings are particularly challenging environments for maintaining patients' dignity.

**Aim:**

The aim of this study was to review previous research to identify nurses' actions that provide humane care and maintain patients' dignity in acute care settings, and to synthesize evidence on how nurses enact dignified humane care.

**Methods:**

This integrative review followed the approach by Whittemore and Knafl and is reported according to the PRISMA guidelines. The databases searched were PubMed, CINAHL, Web of Science, Scopus and PsycInfo. The selected articles were analysed using inductive content analysis.

**Results:**

Thirty‐one articles were included in the review. Nurses' actions demonstrating humane care and maintaining patients' dignity formed five categories: Respecting the patient as a unique individual, Being present to the patient's expressions and experiences, Giving the patient the opportunity to be involved and in control, Protecting the patient's integrity and physical boundaries and Providing individual and appropriate care considering the patient's vulnerability.

**Conclusions:**

Patients' dignity is maintained through everyday nursing actions. Providing humane care and maintaining patients' dignity requires that nurses are genuinely present with the patient to recognise their unique experience.

## 1 Introduction

Nurses' actions that maintain patient dignity are fundamental to good care. However, complex health care settings, such as acute care environments, can present challenges to maintaining patient dignity [1]. Nevertheless, nurses are expected to act in ways that preserve the dignity of patients in their care [2, 3, 4]. Dignity‐related issues have been studied across various clinical settings, for example acute, psychiatric, rehabilitation, elderly and end of life care, where dignity is often understood in terms of the factors that support or threaten a patient's dignity [5, 6, 7]. However, there is a gap in knowledge regarding which specific nursing actions support maintaining patient dignity.

Dignity is recognised as a human right by the United Nations [8]. The concept of dignity has been discussed and defined across different periods and disciplines [9]. Based on the literature, dignity is often approached from two main perspectives [1, 2, 3, 10]. First, dignity is objective, an intrinsic worth that belongs to every human being [2, 3, 8, 10]. Second, dignity is a subjective experience that forms socially, through interaction with other people [1, 2, 10]. Humane care, in turn, forms the basis for nurses' ethics actions and is a requirement for maintaining patient dignity during care [11]. Humane care involves being present with the patient, and it encompasses the elements of social relations, human growth and development, emotional well‐being and physical health [12].

In the context of nursing, dignity is a core concept of nursing ethics [2], and it is the ethos of the humanbecoming paradigm. Dignity is understood as the worth that every person has, simply by being human. Nurses, through their actions in practice, can promote or violate patients' dignity. Honouring dignity manifests as being truly present with an individual and respecting their uniqueness and choices; this is reflected in actions. Nurses are called to be genuinely attentive to their patients' needs in everchanging situations. Perceiving the reality of the patient's experiences honours the patient's dignity [13, 14]. From the patient's perspective, dignity is experienced when they are respected as a valuable individual and when they feel seen, heard and believed in [7]. In acute care settings, dignified care can be defined as care that promotes, and does not undermine, a patient's self‐respect. Dignified care fosters in a patient the sense that they are being treated as a human being [15]. Giving the patient emotional support, responding to their needs and helping them maintain their body image are recognized as elements of dignified care [16]. Violating dignity emerges from patient's experience. A patient's dignity is violated when they do not feel seen, recognized or acknowledged. Dignity is also violated when a patient is subsumed into a group identity and seen based for example sex or race. Invasion of patient's personal space violates dignity, and this might have differences between cultures. One form of dignity violation is humiliation, when a patient is distinguished or separated from a group or from a social norm [17].

Acute health care systems are challenged to provide care for people with complex and long‐term health conditions with many fundamental needs. These needs include being treated with respect and in a manner that preserves their dignity [18]. Respect is regarded as a key element of maintaining patients' dignity. In acute care settings, respect can be defined by nurses' actions [19]. In these environments, patients often feel vulnerable and experience a lack of privacy, which can compromise their dignity [6, 20]. In this study, dignity is conceptualized as the subjective experience of a patient, maintained through nurses' actions when they are truly present with the patient [13]. While patient dignity has been widely discussed in the literature, there is limited evidence describing the actual nursing practices and behaviours used to maintain patients' dignity in clinical care. The aim of this integrative review was to identify nurses' actions that provide humane care and maintain patients' dignity in acute care settings, and to synthesize evidence on how nurses enact dignified humane care.

## 2 Methods

### 2.1 Design

This integrative review was conducted according to an approach described by Whittemore and Knafl and included stages of problem formulation, literature search, data evaluation, data analysis and presenting the results [21]. Reporting was guided by the PRISMA guidelines [22]. The protocol of this review was registered in the International Prospective Register of Systematic Reviews (PROSPERO) (reference: CRD42025640501).

### 2.2 Search Strategy

Data were systematically searched from several databases PubMed, CINAHL, Web of Science, Scopus and PsycInfo from their inception to March 2025. An information specialist from the university library was consulted before performing the searches to confirm the research protocol. Searches were conducted using keywords, Boolean phrases and MeSH terms, when applicable. The search string used was: (“dignified care” OR “patient dignity” OR “humane care” OR “human presence”) AND (nurs* OR caring* OR “patient care” OR “healthcare professionals”) AND (“acute care*” OR hospital* OR patient* OR inpatient*). The inclusion criteria for the selected studies were: nurses (registered nurses, licensed practical nurses, public health nurses) or patients describing nurses' actions of humane care and maintaining patients' dignity in an acute care setting. The language of the selected studies was English. Studies with diverse methodologies were included.

Database hits were exported to the Covidence platform for screening. Duplicates were removed automatically by Covidence. Two independent reviewers screened the articles first by title and abstract and then by full text.

### 2.3 Quality Appraisal

Due to the diversity of the included studies, different Joanna Briggs Institute critical appraisal tools were employed [21, 23]. The methodological quality of the selected studies was evaluated by two independent reviewers. One of the included studies [24] was evaluated using the Checklist for Analytical Cross Sectional Studies. The other included studies were qualitative and were evaluated using the Checklist for Qualitative Research [25]. The scores were used to evaluate the general quality of the selected studies, and no studies were excluded based on the scores.

### 2.4 Data Analysis

The data were analysed using inductive content analysis [26, 27]. First, the selected articles were read several times. Next, relevant expressions describing nurses' actions of humane care and maintaining patients' dignity were condensed. Condensed expressions formed codes. Codes with similar content formed sub‐categories, and sub‐categories were further combined into categories. The sub‐categories and categories were named to reflect the content describing nurses' actions. The first author performed the analysis, and the results were discussed and confirmed with the other authors.

## 3 Results

**FIGURE 1:**
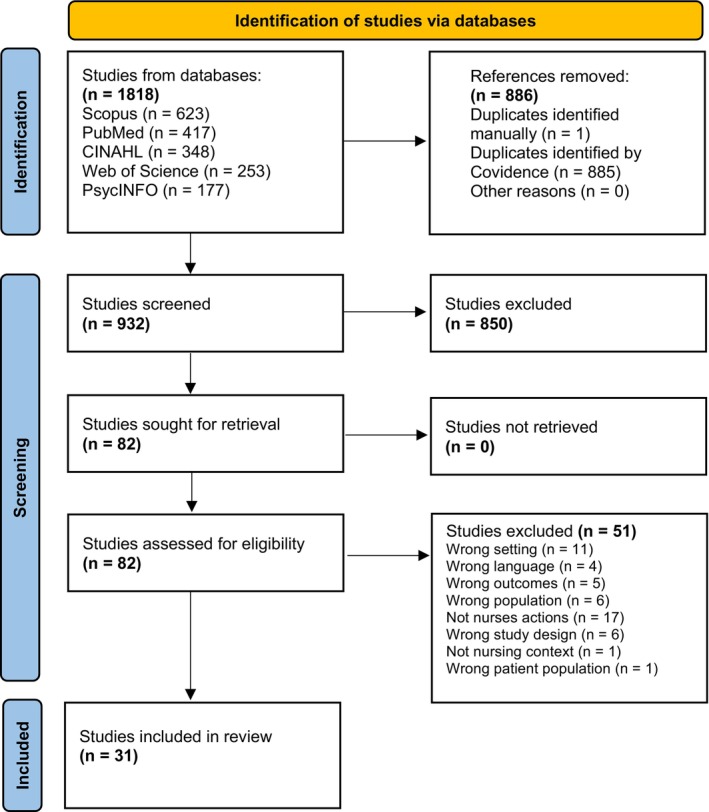
PRISMA flowchart on the screening and selection process.

A total of 1818 articles were identified from the databases, and 886 duplicates were removed. Next, through a title and abstract screening, 850 articles were removed for not meeting the inclusion criteria. Full texts of the remaining 82 articles were read, and 31 articles met the inclusion criteria and were selected for the review (Figure 1).

### 3.1 Description of the Included Studies

The included studies were undertaken in 13 different countries: Iran (*n* = 11), Indonesia (*n* = 4), Sweden (*n* = 4), United Kingdom (*n* = 2), Taiwan (*n* = 2), Turkey (*n* = 1), Ghana (*n* = 1), Spain (*n* = 1), Norway (*n* = 1), Finland (*n* = 1), Pakistan (*n* = 1), Denmark (*n* = 1) and Australia (*n* = 1). Characteristics of the selected studies are presented in Table 1.

**TABLE 1:** Description of included studies.

Author, year, country	Aim	Design, methods, sample	Key findings
Asmaningrum et al., 2019 Indonesia	To explore and compare nurses' and patients' viewpoints of disrespectful behaviours that threaten patient dignity during hospitalized care.	Qualitative design Semi‐structured interviews Inductive content analysis 35 inpatients and 40 registered nurses from medical and surgical wards	Based on the interviews five categories of disrespectful behaviour threatening patients' dignity emerged. Negligence, impoliteness and dismissal were important for both nurses and patients. Inattentiveness was highlighted by nurses and discrimination by patients. Promoting patient centred care can help create a more respectful climate for maintaining patients' dignity.
Asmaningrum & Tsai, 2018a Indonesia	To elicit nurses' perspectives for maintaining patient dignity in Indonesian clinical care settings	Qualitative design Semi‐structured interviews Inductive content analysis 40 nurses in medical and surgical units	According to the interviews, nurses considered three main elements in maintaining patient dignity in clinical care: personalized care, including prioritizing patients and treating them as individuals, compassionate care, including empathizing and providing emotional support, and patient care advocacy, including protecting patient rights and being a representative for the patient. It was considered important to provide dignified care in a manner that is congruent with culture.
Asmaningrum & Tsai, 2018b Indonesia	To gain an understanding of the perspectives of hospitalized inpatients in Indonesia regarding maintaining dignity during clinical care	Qualitative design Semi‐structured interviews Inductive content analysis 35 patients in medical and surgical wards	Four major categories which describing essential elements for maintaining patients' dignity were revealed: responsiveness, respectful nurse–patient relationships, caring characteristics and personalized service. According to this study, besides being clinically competent, nurses must also be culturally competent to provide dignified care.
Aydın Er et al., 2018 Turkey	To explore the opinions and experiences of Turkish patients and nurses about the respectful care of human dignity	Quantitative, descriptive cross‐sectional study Questionnaire Statistical analysis 150 patients and 78 nurses in cardiology, neurology and neurosurgery clinics	According to this study, the most important factor for dignified care was protecting the patients' rights. The nurses also stated that not exposing the patients' body and being respectful of the patients' privacy were important for dignified care. Patients and nurses differed somewhat in the factors they considered important for dignified care.
Bagheri et al., 2012 Iran	To investigate patient dignity and related factors in heart failure patients	Qualitative design Semi‐structured interviews Summative content analysis 22 heart failure patients	Two factors related to heart patients' dignity were identified: Patient/care index and Resources. Patient/care index had sub‐categories intrapersonal features and interpersonal interactions, which included themes describing nurses' actions promoting patients' dignity. The themes were inherent characteristics, individual beliefs, communication, respect, enough information, privacy and authority.
Bagherian et al., 2020 Iran	To evaluate the perception of human dignity among Iranian cancer patients	Qualitative design Semi‐structured interviews Qualitative content analysis method 16 cancer patients in internal medicine wards	Based on the interviews, 3 main themes were determined. The main themes were identified as personal space and privacy, respect for human values and moral support. The results showed the necessity to care for cancer patients in a respectful manner. The key elements in such care were the preservation of patients' personal space and privacy, respect for their values, and the provision of adequate moral support.
Baillie, 2009 United Kingdom	To investigate, in an acute hospital setting, the meaning of patient dignity, how patients' dignity is threatened and how patients' dignity is promoted	Qualitative design Interviews and observation with both patients and ward staff Thematical analysis using a framework approach One surgical ward, 24 patients, 13 members of staff	Being in hospital can compromise patients' dignity. Hospital environments and staff behaviour can influence how patients maintain or lose their dignity. Hospital staff can promote patients' dignity by providing privacy and with interactions that make patients feel valued, comfortable and in control.
Bakhshiarab et al., 2024 Iran	To evaluate nurses' understanding of threats to human dignity in the care of patients with COVID‐19	Qualitative design Semi‐structured interview Content analysis 15 nurses taking care of COVID‐19 patients	According to this study, factors threatening patients' dignity were divided into two categories: unethical behaviour and organizational factors. Identifying these factors can help improve care and contribute to a working environment that upholds patients' dignity.
Borhani et al. 2016, Iran	To explore facilitators and threats to patient dignity in hospitalized patients with heart disease	Qualitative design Semi‐structured interview Conventional content analysis 20 patients and 5 personnel in a coronary care unit	Based on the interviews three themes emerged: care context, holistic safe care and creating a sense of security. These themes were considered important to patients' dignity during care.
Cheraghi et al., 2014 Iran	To explore and understand the common ground of dignified care in two cultural and religious perspectives	Qualitative design Semi‐structured interviews Inductive conventional content analysis 5 Iranian Muslims and 5 Iranian Armenians with a hospital experience	Christian and Muslim patients had common experiences regarding dignity preservation emerging as “exigency of respecting human nobility” and “providing person‐centered care”. In cross‐cultural settings it is essential to recognize the individuality of patients to promote their dignity.
Cheraghi et al., 2015 Iran	To discover the lived experiences of Iranian patients regarding maintaining their dignity in bedside care	Qualitative design, phenomenology semi‐structured deep interviews Diekelmann, Allen and Tanner approach 14 hospitalized adult patients	The findings of this study revealed three main themes of preserving patients' dignity: Exigency of preserving the innate human dignity, Service based on love and kindness, and Dignifying and transcendental professional service. Nurses have an obligation to promote and maintain patients' dignity, which is based on acknowledging the equal inner worth of patients.
Fuseini et al., 2023 Ghana	To explore older adults' perspectives about dignity and dignified nursing care during acute hospitalization in Ghana	Qualitative design Semi‐structured interviews Reflexive thematic analysis 20 patients in medical and surgical wards	Four themes were identified: Effective nurse–patient communication, Maintaining patients' privacy, Respectful and compassionate care provision and Providing quality and safe care. Effective communication, patients' education about their health condition, shared decision making, privacy, cultural beliefs and environmental factors are important when providing dignified nursing care.
Gustafsson et al., 2013 Sweden	To illuminate the meaning of the maintenance of patient dignity in forensic care	Qualitative design Focus group interviews Phenomenological hermeneutic analysis 7 forensic care nurses	Three themes emerged: to protect the patient, to respect the patient and to meet patients in human brotherhood. Dignity cannot be given to the patient, but when nurses support and respect patients, the patients can keep and maintain their dignity.
Gustafsson et al., 2014 Sweden	To describe nurses' experiences of violation of patient dignity in clinical caring situations in involuntary psychiatric hospital care	Qualitative, hermeneutic Group interviews once a month for 9 months Hermeneutic method 15 nurses in psychiatric wards	According to this study, seven themes of nurses' experiences of violations of patient dignity emerged: patients not taken seriously, patients ignored, patients uncovered and exposed, patients physically violated, patients becoming the victims of others' superiority, patients being betrayed, and patients being predefined. Focusing on ethical problems in care is important, especially when taking care of vulnerable groups of patients.
Heijkenskjöld et al., 2010 Sweden	To understand how nurses experience patients' dignity in Swedish medical wards	Qualitative, hermeneutic individual texts from the nurses hermeneutic text interpretation 12 nurses at medical wards	With their actions nurses can either protect or violate patients' dignity. Patients' dignity is maintained when nurses treat patients as fellow human beings. Dignity is violated if nurses treat patients as objects.
Jamalimoghadam et al., 2019 Iran	To explore hospitalized adolescents' perceptions of dignity	Qualitative design Unstructured interviews Conventional qualitative content analysis 13 adolescents aged 12–18 years in medical and surgical wards	Four themes emerged: protection of personal privacy, protection of autonomy, respect for identity, and intimate communication. Human dignity is a core value in nursing, and nurses should be aware of it on paediatric wards.
Lin & Tsai, 2011 Taiwan	To understand how nurses in Taiwan maintain patients' dignity in clinical practice	Qualitative design In‐depth interviews content analysis 30 nurses in internal or surgical wards	Based on the interviews five themes emerged: respect, protecting privacy, emotional support, treating all patients alike and maintaining body image.
Lin et al., 2011 Taiwan	To explore dignity in care from patients' perspectives in Taiwan	Qualitative design In‐depth interviews Content analysis 40 patients in surgical and medical wards	Six themes describing preservation of patients' dignity were identified: sense of control and autonomy, being respected as a person, avoidance of body exposure, caring from the nursing staff, confidentiality of disease information and prompt response to needs. Patients were mostly satisfied with how their dignity was maintained.
Lindwall & von Post, 2014 Sweden	To obtain an understanding of what nurses experienced as dignity in surgical practice	Qualitative design 49 written stories Hermeneutical text interpretation 11 surgical practice nurses	According to this study, patients' dignity was preserved when healthcare professionals allowed patients to tell their stories and allowed themselves to get close to a patient and in turn received the patient's trust. Patients' dignity was violated when healthcare professionals behaved rudely towards the patient, acted as if the patient was invisible or humiliated the patient at the end of life.
Manookian et al., 2014 Iran	To explore the factors that influence, promote or compromise patient dignity	Qualitative design Semi‐structured interviews Inductive content analysis 14 patients with experience in acute care hospitalization	Three themes describing factors influencing patients' dignity were explored: persona, communication behaviours and staff conduct. Recognizing and focusing on these factors will help provide more dignified care.
Martin‐Ferreres et al., 2019 Spain	To explore the delivery of dignified care by professional nurses	Qualitative design, ethnography Observation and semi‐structured interviews Inductive method 27 for observation, 20 for interviews nurses in internal medicine ward	Two themes emerged based on the data: delivering dignified care and factors influencing the delivery of dignified care. There was a discrepancy between nurses' perceptions of the care they offered and what they actually did.
Matiti & Trorey, 2008 United Kingdom	To explore patients' expectations regarding the factors that contribute to the maintenance of their dignity while in hospital	Qualitative, phenomenological hermeneutic approach Interviews Phenomenological hermeneutic approach 102 patients in medical, surgical and orthopaedic wards	Based on the interviews, six themes emerged: privacy; confidentiality; communication and the need for information; choice, control and involvement in care; respect and decency; and forms of address. Patients expressed satisfaction with some aspects related to maintaining dignity but also several areas where their needs were not met and how they would like them to be met.
Moen & Nåden, 2015 Norway	To acquire knowledge of what contributes to maintaining and promoting the dignity of intensive care patients	Qualitative, phenomenology Individual interviews Phenomenological analysis strategy 7 patients who had been treated in an Intensive Care Unit	According to the interviews, five themes concerning dignity for the intensive care patient emerged: being heard and seen, letting the carers take over, frustration about inability to speak, being respected and feeling violated. For dignified care, important elements were being seen and heard, and having one's wishes and needs attended. It was considered essential to be met with respect and to provide personal and individual nursing. Feelings of being helpless and being treated as an object threatened dignity.
Mohammadi et al., 2023 Iran	To explore the concept of dignity in patients who attempted suicide, from the perspective of psychiatric nurses	Qualitative design Semi‐structured individual interviews and field notes Conventional content analysis 20 psychiatric nurses	Based on the study, three main themes emerged: respect for personal identity, management of psychological tension and compassion‐focused therapy. Patients who have attempted suicide should be cared for in a supportive environment with compassionate and respectful behaviour to maintain their dignity.
Namadi et al., 2022 Iran	To explain threats to the dignity of hospitalized COVID‐19 patients	Qualitative design Semi‐structured interviews Conventional content analysis 21 patients who had been hospitalized for COVID‐19	The interviews revealed three main categories of threats to the dignity of COVID‐19 patients. The main categories included facing imposed conditions, facing unprofessional performance, and ineffective communication. Many threats are controllable, and identifying them can help maintaining patients' dignity.
Nyholm & Koskinen, 2017 Finland	To highlight, from a caring perspective, how nurses in an intensive care setting understand patient dignity, what threatens patient dignity and how nurses can safeguard patient dignity	Qualitative design Survey questionnaire containing open questions Hermeneutic reading and text interpretation 25 nurses working at an intensive care unit	According to this study, nurses recognize patients' absolute dignity. The interpretation of the text revealed three patterns: dignity as a fundamental ethical value in caring, threats against patient dignity and safeguarding patient dignity. These patterns describe nurses' understanding of intensive care patients' dignity.
Rafiq et al., 2021 Pakistan	To explore nurses' perceptions of the dignity of intubated patients in the ICU	Qualitative design In‐depth individual interviews Content analysis 14 intensive and critical care nurses	Four major themes emerged: two sides of contemporary nursing practice, benefits of dignified nursing care, challenges to the dignity of intubated patients and strategies for promoting the dignity of intubated patients. Dignified care is essential for quality nursing care and patient well‐being in high‐dependency units.
Rasmussen & Delmar, 2014 Denmark	To describe the characteristics of the importance of dignity perceived by four surgical patients at a university hospital in Denmark	Qualitative, hermeneutic phenomenology Semi‐structured interviews Thematic analysis 4 surgical patients	The thematic analysis of the interviews led to one basic theme: To be an important person. This was illustrated by several sub‐themes: Being a co‐player, Over exposure and To swallow the bitter pill. Patients' dignity is a complex interaction of several factors. Patients have a clear perception of factors that affect dignity, expectations and how to maintain dignity.
Tehranineshat et al., 2021 Iran	To identify and describe burn patients' dignity based on the experiences of nurses, family caregivers, and burn patients	Qualitative design Semi‐structured interviews Conventional content analysis 14 nurses, 6 family caregivers, and 5 patients in a burn hospital	Based on the interviews, three main themes were extracted: empathic communication, showing respect, and providing comprehensive support. Effective communication, spending time with patients and attending to patients' repetitive requests are important elements of care to maintain patients' dignity.
Walsh & Kowanko, 2002 Australia	To uncover patients' and nurses' perceptions of dignity, formulate a definition of dignity based on the experience of patients and nurses, and identify nursing practices that maintain or compromise patient dignity	Qualitative design, phenomenology Unstructured interviews Interpretative hermeneutic approach 9: 4 nurses and 5 patients in an acute hospital	Similar elements emerged from both nurses' and patients' interviews. Nurses described elements of respect, privacy, control, advocacy and time. The patients described themes of respect, privacy, control, choice, humour and matter‐of‐factness. Patients felt that their dignity was maintained when they were given choices and when they sensed they had control over their care.
Wulandari & Rochmawati, 2025 Indonesia	To explore the experiences of nurses in providing dignified care for patients with life‐limiting illnesses	Qualitative design Semi‐structured interviews Phenomenological data analysis method 15 nurses who provided care for patients with life‐limiting illness	Three themes emerged: Establishing a therapeutic environment, Respecting the humanity of patients and strengthening the spirituality of patients. Creating a therapeutic environment is essential for dignified care. A therapeutic environment can be created by building trust, providing compassionate care and motivating patients. Humanized care and spirituality are important components of providing dignified care.

One of the studies [24] utilized a quantitative, cross‐sectional approach, while the other 30 studies employed a qualitative approach. Concerning methodology, one study used a questionnaire and statistical methods [24], and three studies used written materials and a hermeneutic text interpretation [28, 29, 30]. The other 27 studies were based on interviews with one combining interviews with observation [31]. The interviews were analysed using content analysis [32, 33, 34, 35, 36, 37, 38, 39, 40, 41, 42, 43, 44, 45, 46, 47, 48], thematic analysis [20, 31, 49], the Diekelmann, Allen and Tanner approach [50], hermeneutic analysis [51, 52, 53, 54] or phenomenological analysis [55].

The included studies were conducted in various acute hospital environments. In some cases, there were participants from different wards. One study was conducted with participants who were adolescents aged 12–18 years [40]. All the other studies involved adult participants. Three of the studies were conducted in psychiatric environments [45, 51, 52] and three in intensive care units [30, 47, 55]. The other studies were conducted in various somatic wards.

### 3.2 Findings

Nurses' actions demonstrating humane care and maintaining patient dignity formed five categories: Respecting the patient as a unique individual, Being present and attentive to the patient's expressions and experiences, Giving the patient the opportunity to be involved and in control, Protecting the patient's integrity and physical boundaries and Providing individual and appropriate care considering the patient's vulnerability (Table 2).

**TABLE 2:** Categories and sub‐categories of nurses' actions of humane care and maintaining patients' dignity.

Category	Sub‐category
Respecting the patient as a unique individual	Supporting patient's individuality Being sensitive to cultural and religious aspects Communicating respectfully
Being present to the patient's expressions and experiences	Encountering the patient in shared humanity Showing empathy and compassion Taking the patient seriously Supporting the patient to be empowered Providing time and presence
Giving the patient the opportunity to be involved and have a sense of control	Supporting patient's autonomy Delivering truthful information Securing of informed consent as a precondition for care
Protecting the patient's integrity and physical boundaries	Upholding patient's right to confidentiality Protecting privacy of the body Providing the patient a private space
Providing individual and appropriate care considering the patient's vulnerability	Responding to patient's needs Delivering respectful and competent care Treating patients equally Being patient's advocate

#### 3.2.1 Respecting the Patient as a Unique Individual

Respecting the patient as a unique individual comprised three sub‐categories: Supporting patient's individuality, Being sensitive to cultural and religious aspects and Communicating respectfully.

Nurses' actions supporting patients' individuality contribute to the maintenance of patient dignity. Patients valued being called by name or a familiar nickname. Patients expressed that, when nurses used the patient's name instead of bed number for example, they felt that the nurses saw them as individuals [33, 39, 40, 41, 46, 53, 56]. Also, when nurses gave the patients a possibility to wear their own clothes and other personal items, it made them feel recognized as an individual; wearing hospital clothes made patients feel anonymous [45, 49, 53]. Individuality was also supported when patients had an opportunity to act according to their normal habits, for example, following their normal sleep pattern [35, 40, 45, 49, 53]. Nurses treating patients in an age‐appropriate manner reinforced the patient's sense of individuality and dignity; for example when adolescents were not treated like young children [28, 40, 52].

Nurses being sensitive to a patient's cultural or religious identity promoted the patient's dignity. In some cases, respecting religious tradition calls for nurses to be of the same sex as the patient. Being cared for by a nurse of the opposite sex can be experienced as a violation of the patient's dignity [36, 40, 46, 47, 48]. Respecting other religious habits, beliefs and values of patients is also essential for maintaining patients' dignity. In practice, this can be performed by paying attention to religious needs, supporting religious activity or considering and being sensitive to a patient's values when providing care [24, 38, 39, 41, 43, 45, 48, 56]. Some cultural norms require that only a spouse or a close relative can see a person naked; otherwise, dignity is compromised. In these situations, nurses permitting family members to be involved in patient care was seen as important [20].

Communicating respectfully is also part of maintaining patient dignity. Effective communication in nursing involves not only the content of the message but also the manner in which it is conveyed and the communicative behaviours exhibited by nurses during interactions with patients. Using clear and appropriate language, without too many medical terms and taking into account patient's mother tongue promotes patient's dignity [20, 33, 34, 39, 41, 43, 44, 53]. Nurses also support patient dignity through non‐verbal communication including eye contact, smiling and gestures and acting consistently with their words [29, 33, 34, 39, 43, 46, 48, 49, 52, 53]. Support for patient dignity was also experienced when patients felt heard by nurses [24, 28, 29, 30, 33, 34, 38, 39, 40, 41, 42, 45, 49, 50, 52, 53, 56].

#### 3.2.2 Being Present and Attentive to the Patient's Expressions and Experiences

Being present and attentive to the patient's expressions and experiences comprised five sub‐subcategories: Encountering the patient in shared humanity, Showing empathy and compassion, Taking the patient seriously, Supporting the patient's empowerment and Providing time and presence. These sub‐categories reflect nurses' actions that ensure that patients feel seen and heard.

Encountering the patient in shared humanity was reflected in nurses' actions in various ways. For example, when nurses introduced themselves when meeting a patient or before beginning care procedures, it promoted patients' sense of dignity. Such introductions made patients feel that nurses encountered them as valuable, fellow human beings [33, 39, 49, 53]. Nurses' use of humour or playfulness with patients was also experienced as an action of shared humanity [29, 31, 54, 55]. When nurses went above and beyond their routine duties, performing actions not necessarily required, such as spending extra time with patients, patients felt genuinely recognized in their humanity [51, 55]. Encountering the patient in shared humanity also emerged when nurses showed a more humane side of themselves, rather than only maintaining a professional demeanour [39, 52].

Nurses' actions of showing empathy and compassion were recognized as promoting patients' dignity. Empathy was demonstrated through nurses' actions, particularly when they conveyed an understanding of patients' emotional states and the circumstances they were experiencing [28, 33, 35, 36, 42, 44, 45, 46, 48, 49, 50, 56]. Nurses demonstrated compassion when they offered comfort and emotional support to patients [20, 29, 33, 39, 43, 48, 50].

Taking the patient seriously without questioning their feelings or complaints also supported patient dignity. Nurses demonstrated active listening and trust in patients by acknowledging the credibility of what they said and showing a willingness to engage attentively in the conversation [28, 30, 52, 56].

Being present to patient's expressions and experiences was reflected in nurses' actions in supporting the patient's empowerment, including when nurses provided encouragement and motivation to patients [33, 34, 53, 55]. Empowerment was promoted when nurses tried to teach patients coping skills for various situations, as well [42, 45, 51]. Supporting empowerment also emerged in nurses actions when they helped patients maintain hope [36, 56] In addition, nurses tried to reduce factors that caused distress to patients [33, 45].

Providing time and presence was observed in nurses' actions when nurses worked without rushing and with enough time to do their tasks. Patients felt respected when nurses took time to listen to them even when they were busy [24, 29, 33, 34, 38, 40, 43, 48, 49, 52, 53, 54].

#### 3.2.3 Giving the Patient the Opportunity To Be Involved and in Control

Giving the patient the opportunity to be involved and in control formed through three sub‐categories: Supporting patient autonomy, Delivering truthful information and Securing informed consent as a precondition for care.

Nurses supported patient autonomy when they gave patients a chance to be involved in decision‐making in their care, for example, by asking a patient what they wanted or how they wanted to be treated and giving patients choices [20, 28, 29, 30, 37, 39, 40, 42, 48, 49, 51, 53, 54, 55, 56]. Having choices gave the patients a sense of involvement and control [49]. In addition, nurses respecting and protecting a patient's right to decide was appreciated [33, 36, 40, 47].

According to the selected studies, nurses' delivery of truthful information helped to maintain patients' dignity. Nurses provided patients with sufficient honest information about their situation, as often as needed [20, 24, 30, 31, 32, 33, 35, 39, 41, 46, 48, 49, 50, 53, 56]. If a patient had questions, nurses were willing to answer them [28, 50, 55] Nurses' also provided educational information to patients considering their situation [38, 48, 56].

Securing informed consent as a precondition for care gave patients a sense of control in their own care. Before performing any procedure, nurses provided information to the patients about what would happen and asked for their consent [24, 28, 31, 34, 37, 42, 47, 55, 56]. Nurses obtaining patients' consent before documenting, for example taking a photo, was also considered respectful [56]. In addition, informed consent was sought when a patient was asked to participate in research or when students were involved in care [20, 24].

#### 3.2.4 Protecting the Patient's Integrity and Physical Boundaries

Protecting the patient's integrity and physical boundaries comprised three sub‐categories: Upholding the patient’ right to confidentiality, Protecting bodily privacy and Providing the patient a private space.

Nurses upheld patients' right to confidentiality. Nurses avoided talking about private medical information in the presence of others. When possible, they ensured private settings for discussions [24, 33, 36, 38, 41, 42, 44, 45, 47, 53, 54, 56]. When privacy was not possible, nurses lowered their voice during conversations [41, 53]. In addition, nurses were aware that a patient's health condition could be exposed if someone else observed the patient's medication [52]. However, patients trusted nurses to not their personal information or stories with anyone [29, 40].

Nurses protected bodily privacy, which promoted patient dignity. Nurses tried to avoid unnecessary body exposure, revealing only the body parts necessary for treatment [24, 31, 36, 41, 42, 44, 49, 51, 53, 54, 55]. Nurses protect patients' privacy using curtains and doors to prevent body exposure and any unwanted gaze [20, 30, 36, 41, 42, 44, 49, 53, 54, 55, 56]. Recognizing that hospital gowns can lead to unintended body exposure, nurses encouraged patients to wear personal clothing to maintain comfort and dignity [31, 49, 54].

Despite the challenges of a hospital environment, nurses tried to provide patients with private spaces. Privacy included both physical and emotional aspects. Physical privacy involved using doors or curtains to provide the patient with a private space [30, 31, 33, 36, 37, 40, 42, 47, 48, 49, 53, 54]. Nurses knocked on the door before entering a patient's private space [44, 53]. Patients' bed areas were respected as private spaces [54]. Privacy issues were also considered when patients used the toilet or shower [20, 54]. In addition, nurses sought to provide patients with emotional privacy, creating spaces where patients could express their emotions confidentially [38, 54]. Nurses made efforts to avoid disturbing patients' emotional privacy by not being overly curious, as well [29].

#### 3.2.5 Providing Individual and Appropriate Care Considering the Patient's Vulnerability

Providing individual and appropriate care considering the patient's vulnerability was formed based on four sub‐categories: Responding to the patient's needs, Delivering respectful and competent care, Treating patients equally and Being the patient's advocate.

Nurses' responsiveness to patients' needs promoted the maintenance of patient dignity. Patients felt respected and valued when nurses responded promptly and they did not have to wait for help. Meeting patients' needs without delay helped maintain patient dignity [20, 24, 33, 34, 38, 39, 41, 46, 48, 50, 51, 53, 54]. Likewise, when patients felt that their needs were prioritized, their dignity was promoted [33, 34, 56]. Sometimes patients experienced their needs being met without them even asking [32, 34, 55].

Respectful and competent care was also understood as part of preserving patient dignity, for example, when nurses were kind and helpful when treating patients [30, 33, 34, 38, 43, 49]. In addition, nurses having patience [33, 43], being respectful (20,29–31,33,35,43,44,54,55)and showing professional commitment and responsibility [30, 35, 43, 46, 53] when delivering care made patients felt valuable and well cared for [30, 33, 43]. Patient dignity was promoted when nurses treated patients equally, without discrimination [30, 32, 33, 42, 50, 56] and by not giving some patients more attention than others [24, 32, 33, 35, 36, 39, 48, 56].

Nurses promoted patient dignity by being an advocate for patients' dignity. Nurses sought to prevent situations in which a patient's dignity could be compromised [52, 54]. When a patient could not speak for themselves, nurses took measures so that the patient's dignity was maintained. For example, nurses ensured patients' dignity at the end of life [37, 53, 54]. Avoiding unnecessary or futile treatment was also seen as protecting patient dignity [24, 30, 54]. In addition, nurses tried to avoid stigmatizing patients [37, 45].

## 4 Discussion

This study provides novel insights into nurses' actions that maintain patient dignity in different acute care settings. These findings build on previous research focusing on patients' experiences in elements seen as supportive of or threatening to patient dignity. Nurses' actions in everyday practice promote patient dignity when they prioritise the patient's humanity and consider the patient's experience. Patients construct meaning in constantly changing situations, regardless of age or health conditions, but as part of an ongoing processes of being an individual [57]. Nursing care itself is complex and uncertain by nature. This challenges nurses to strive to understand the patients' experience, instead of routine care [3] Truly being present with a patient enables nurses to recognise the patient's experience and deliver care in ways that maintain the patient's dignity.

This study describes nurses' actions when they are truly present with the patient. Such presence requires humane care and being close to the patient. Acute care settings involve opportunities for and limitations to nurses being truly present with patients. According to the findings of this study, patient experiences of the preservation of dignity in nursing practice in acute care settings were similar regardless of a patient's age or health condition. For example, adolescents reported experiences of dignity maintenance akin to those of adults; adolescents simply used different wording to describe their experiences [40]. Likewise, the experiences from psychiatric settings were similar to those experiences from somatic settings, again, with varied wording [45, 51, 52]. Dignity is inherent to every human being, but it is experienced in a subjective, everchanging way. Despite this, the results of this study indicate that, across different age and patient groups, experiences concerning dignity are similar, reflecting a shared experience of being human. However, the shared experience of maintaining dignity in care requires further research.

In this study, respectful and polite communication, giving emotional support, providing time and truthful information, responding to patients' individual needs and providing respectful and competent care were recognized as nurses' actions that help maintain patient dignity in acute care settings. Previous studies have identified respect as one of the key elements of dignity [7, 9, 20]. Also, from the humanbecoming perspective, respectful behaviour and respecting the uniqueness of individuals and their choices promotes patient dignity [13, 14]. In acute care settings, respect can be defined through nurses' actions such as polite behaviour, listening to patients, taking care of emotional, physiological and informational needs, responding to patients' wishes and giving time to patients [19]. This is confirmed in the findings of this study. In practice, it is possible to have too little or too much respect. A lack of respect can lead to violations of the patient's dignity. An excessive amount of respect can result as sycophancy [3]. Determining an appropriate level of respect is difficult. For this, humane care and being truly present for the patient are needed to understand their experience and to act the way that patients' experience concerning their dignity is being respected.

The results of this study indicate that nurses maintain patients' dignity by being present and attentive to patients' experiences and expressions and providing patients with the opportunity to be involved in having a sense of control of their care. Being present requires a level of closeness with the patient, which refers to humane care [12]. From this perspective, nurses' being present with patients can be seen as a core element of nursing practice. Through truly being present, nurses can recognize the meanings that patients create from their experiences. Also, based on previous research, patients experience dignity when they feel seen, listened to and believed in [7]. Seeing, listening and believing can be understood as manifestations of nurses being present with their patients as they create meanings from their experiences [13, 14]. When the patient is not seen and feels that they have not been recognized or acknowledged, is a violation of patient's dignity [17] Providing good care aims enhancing patient's dignity and also succeeds in realizing this in practice [4] This highlights the importance of humane care as a precondition for maintaining patient's dignity. However, maintaining dignity is not only about nurses doing actions; rather, it is about actions based on nurses' perceptions when they are delivering humane care. Patient's dignity cannot be maintained without being close to and without seeing the patient.

Acute health care settings are challenging environments for maintaining patient dignity. Today's health care is characterized by efficiency and increased productivity, while patients often present with more complex health problems. Acute care settings are known as environments where patients and their dignity are particularly vulnerable [6, 7, 18]. This highlights the importance of protecting patients' integrity and physical boundaries and providing individual and appropriate care considering patients' vulnerability. Especially in acute care settings, where patient dignity might be difficult to maintain, it is crucial that nurses promote dignity through their actions. Further research is needed on how nurses' moral distress and emotional resilience affect their actions to maintain patient dignity in high‐pressure acute care settings.

## 5 Methodological Considerations

This review was conducted systematically following the five stages described by Whittemore and Knafl [21] and is reported according to the PRISMA guidelines [22]. An information specialist was consulted before performing the searches to guarantee the robustness of search terms and sentences, but it is possible that, due to the English language restriction, some relevant studies may have been missed. The articles were selected by two independent reviewers; in cases of any discrepancies, the third reviewer was consulted to reach consensus. The data were analysed with inductive content analysis, and the findings were discussed within the research team to ensure trustworthiness. No studies were excluded based on quality appraisal to capture all relevant information. However, the varying quality of the selected studies is a limitation for this study.

## 6 Conclusions

In acute care settings, patient dignity is maintained through nurses' everyday actions. Humane care and maintaining patients' dignity do not require a specific intervention. Humane care manifests in nurses being truly present with their patients and encountering them as individuals in shared humanity when delivering care. Truly seeing the patient maintains patient's dignity and not seeing and acknowledging the patient and their experiences violates dignity. Dignity is an individual, changing experience. Each patient has their own experience concerning their dignity and it is not possible to predetermine how much or what kind of actions each patient needs for their dignity to be maintained. Consequently, humane care and genuine presence are required for nurses to deliver actions that maintain patient dignity and to appropriately respond to patients' needs in everchanging situations.

## Author Contributions

**Elina Paala:** conceptualisation, methodology, literature search, full text review, quality appraisal, data collection, data analysis, draft preparation, revision and editing, final submission. **Sanna Koskinen:** conceptualisation, methodology, data analysis, draft preparation, revision and editing, supervision. **Minna Stolt:** conceptualisation, methodology, full text review, quality appraisal, data analysis, draft preparation, revision and editing, supervision.

## Funding

The authors have nothing to report.

## Conflicts of Interest

The authors declare no conflicts of interest.

## Data Availability

The data that support the findings of this study are openly available in the databases PubMed, CINAHL, Web of Science, Scopus and PsycInfo.
